# Fibroblast Growth Factors in the Management of Acute Kidney Injury Following Ischemia-Reperfusion

**DOI:** 10.3389/fphar.2020.00426

**Published:** 2020-04-08

**Authors:** Lian-Cheng Deng, Tahereh Alinejad, Saverio Bellusci, Jin-San Zhang

**Affiliations:** ^1^ Center for Precision Medicine, The First Affiliated Hospital of Wenzhou Medical University, Wenzhou, China; ^2^ Institute of Life Sciences, Wenzhou University, Wenzhou, China

**Keywords:** fibroblast growth factors, ischemia-reperfusion injury, acute kidney injury, protection, therapy

## Abstract

Ischemia-reperfusion injury (IRI), which is triggered by a transient reduction or cessation of blood flow followed by reperfusion, is a significant cause of acute kidney injury (AKI). IRI can lead to acute cell death, tissue injury, and even permanent organ dysfunction. In the clinic, IRI contributes to a higher morbidity and mortality and is associated with an unfavorable prognosis in AKI patients. Unfortunately, effective clinical drugs to protect patients against the imminent risk of renal IRI or treat already existing AKI are still lacking. Fibroblast growth factors (FGFs) are important regulators of key biological and pathological processes, such as embryonic development, metabolic homeostasis and tumorigenesis through the regulation of cell differentiation, migration, proliferation and survival. Accumulating evidence suggests that altered expression of endogenous FGFs is associated with IRI and could be instrumental in mediating the repair process. Therefore, FGFs have been proposed as potential biomarkers in the clinic. More importantly, exogenous FGF ligands have been reported to protect against renal IRI and display promising features for therapy. In this review, we summarize the evidence and mechanisms of AKI following IRI with a focus on the therapeutic capacity of several members of the FGF family to treat AKI after IRI.

## Introduction

Acute kidney injury (AKI) is a syndrome and significant cause of death among hospitalized patients. AKI is characterized by a rapid decline in renal function and predisposes the transition of AKI patients to chronic kidney disease (CKD) and end-stage renal disease (ESRD) (Chawla et al., 2014). Ischemia-reperfusion injury (IRI) is the primary cause of AKI worldwide related to different clinical circumstances such as shock, low cardiac output, and organ transplantation (Park et al., 2017; Ronco et al., 2019). Under these conditions, the reestablishment of blood flow after transient obstruction of circulation leads to renal injury. A multinational, multicenter study on critically affected patients confirmed the prevalence of AKI (5.7% of the patients included in the study) and association with a high mortality rate (Uchino et al., 2005). Another independent study shows an even higher percentage of patients admitted to hospital with such a complication, 10–15% of all hospitalisations (Al-Jaghbeer et al., 2018), the number is as high as 50% in patients in the intensive care unit (ICU) 50% (Hoste et al., 2015).

**Figure 1:**
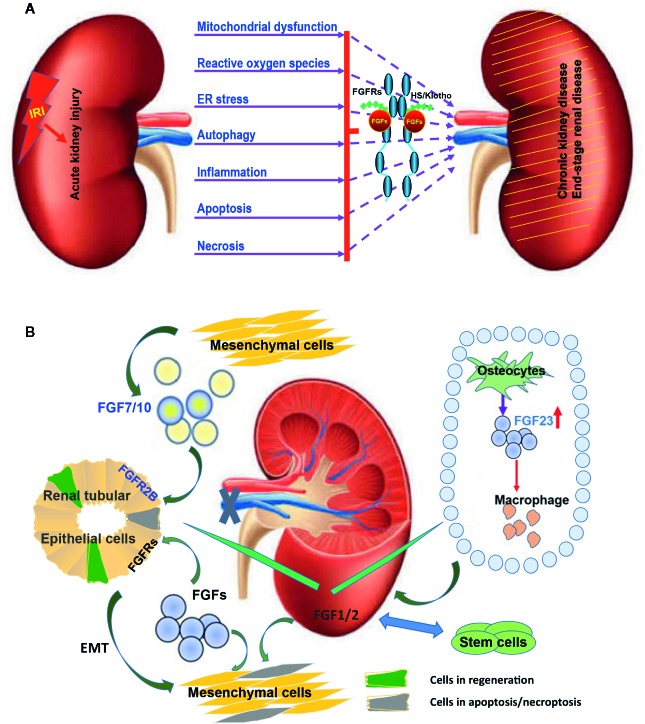
Multiple FGFs are involved in the etiology and pathogenesis of AKI after IRI. **(A)** Diagram summarizing key biological processes underlying the etiology and pathogenesis of AKI. The interactions of these complex disease mechanisms can lead to CKD and ESRD. FGFRs can be activated by endogenous FGFs and co-ligands following IRI. Exogenous recombinant FGFs such as FGF2 and FGF10 can protect again IRI and inhibit the transition of AKI to CKD and ESRD *via* regulating this complex pathogenesis and repair process. **(B)** The expression of several FGFs including FGF1/2/7/10 is induced upon IRI and is capable of promoting tubular epithelial cell proliferation through a paracrine effect. Furthermore, FGF1/2 mediated activation of FGFRs can inhibit the apoptosis of tubular epithelial cells and promote the transformation of tubular epithelium to mesenchymal cells. Exogenous stem cells can ease IRI by producing FGF1/2. Increased FGF1/2 can further support the survival of stem cells. FGF23 is produced by osteoblasts in bone in response to local and systemic factors and targets the kidney to create multiple endocrine networks. FGF23 also impacts macrophage infiltration through adjusting the immune system after IRI.

The disease mechanisms underlying the etiology and pathogenesis of AKI are complex and include mitochondrial dysfunction, reactive oxygen species (ROS), endoplasmic reticulum stress (ERS), autophagy, inflammation, apoptosis, and necrosis (Verma and Molitoris, 2015; Duann et al., 2016; **Figure 1A**). Even though renal IRI is known to be the predominant cause of morbidity and mortality, no effective treatment is currently available. Therefore, much attention has been dedicated to seeking novel therapeutic strategies for AKI. Intense research efforts using animal models have shed light on the pathophysiology of AKI. It has been reported that the expression of Fibroblast growth factors (FGFs) and their receptors (FGFRs) are increased in the context of AKI (Wai et al., 2013; Tan et al., 2017). Furthermore, AKI is more severe upon FGFR deficiency or blockade of its signalling (Villanueva et al., 2008; Xu and Dai, 2017). Some studies demonstrated that renal function recovered after administering various medications, including growth factors and cell transplantation (Ichimura et al., 1996; Patel et al., 2012). These results show that FGFs and their receptors are important for AKI.

In the context of IRI, several growth factors have been reported to exhibit protective effects as well as therapeutic potential as they not only prevent the damages from occurring, but also improve functional recovery after the damages are done (**Figure 1A**, **Table 1**). New functions of FGFs have recently emerged. Evidence for FGF1, FGF2, FGF7, and FGF10 to trigger mitogenic and antiapoptosis activities correlates with their ability to enhance the survival and outgrowth of various kidney cell types, such as collective cells, tubule cells, and glomerular cells. Progress has also been made in understanding the roles and associated mechanisms of FGFs in AKI (**Figure 1B**).

**Table 1:** Fibroblast growth factors (FGFs) directly involved in regulating acute kidney injury (AKI).

FGF Subfamily	FGF ligand	Mode of action	Pathophysiological function
**FGF1**	**FGF1**	Autocrine and paracrine	Inhibition of neutrophil infiltration (Cuevas et al., 1999); Antiapoptosis and regeneration (Fu et al., 2004; Weng et al., 2004)
	**FGF2**	Paracrine	Attenuating mitochondrial damage and proinflammatory response (Tan et al., 2017) reduce renal damage and participate in the regeneration (Villanueva et al., 2006)
**FGF7**	**FGF7**	Paracrine	Promote bladder progenitor proliferation (Vinsonneau et al., 2010)
	**FGF10**	Paracrine	Antiapoptosis and inflammatory response; suppressing excessive autophagy and ER stress (Tan et al., 2018; Tan et al., 2020)
**FGF19**	**FGF23**	Endocrine	Biomarkers for injury and prognosis; amplify myofbroblast activation; potential target of therapy (Leaf et al., 2017; Smith et al., 2017b; Leaf et al., 2018; Volovelsky et al., 2018; Christov et al., 2019, and references within)

Here, we will summarize the available data on the roles and known mechanisms of FGFs in the pathogenesis, prevention, and repair of AKI with a focus on IRI. The discussion will first provide a succinct overview of FGF/FGFR signalling specificity and function, followed by a detailed summary of the published work on the roles of FGFs/FGFRs in the AKI, impact of endogeneous and recombinant FGFs as prevention and therapeutic measures for the IRI, pathophysiological processes and conclude with a highlight for future research to better understand underlying mechanism and FGFs in AKI disease and provide viable strategies to prevent IRI or avert progression to CKD.

## A Succinct Overview of FGF Ligands and Their Receptors

**Figure 2:**
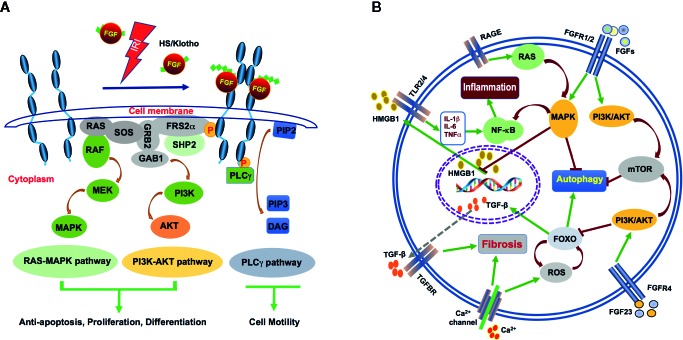
Mechanism of FGFs signalling during AKI after IRI. **(A)** FGFs interact with FGFRs with HS (and klotho for FGF23) as cofactor after IRI. The interactions induce activation of the RAS-MAPK, PI3K-AKT, and PLCγ pathways. These pathways mediate antiapoptosis, differentiation, proliferation, and cell motility. **(B)** HMGB1, a nuclear transcription factor protein is released upon IR injury. Circulating HMGB1 can interact with TLRs to promote inflammatory cytokine secretion. Increased IL-1β, IL-6 and TNF-α in turn, activate NF-κB and further enhance inflammation. FGF2 may inhibit inflammation through robust protection of renal tubular cells from IR-induced apoptosis and subsequent release of HMGB1. FGF2 and FGF10 may inhibit autophagy *via* activation of PI3K/AKT and MAPK signalling. On the other hand, the endocrine FGF23 binds FGFR4 to activate the calcium channel and contribute to renal fibrosis. A large amount of calcium ion influx results in ROS activation, which also leads to increase in TGF-β expression and its down stream signalling to promote fibrosis.

In mammalians, the FGF system consists of 18 ligands signalling through their specific FGFRs (Goetz and Mohammadi, 2013; Ornitz and Itoh, 2015, **Figure 2A**). The molecular weight of vertebrate FGFs ranges from 17 to 34 kDa and the proteins consist of a central core of 140 amino acids and 12 antiparallel β chains. The sequence similarity among different members is between 30% and 60%. FGFs are structurally related and functionally relevant, they elicit a redundant but also distinct repertoire of biological activities. FGFs are further divided into several subfamilies based on their sequence identity, receptor binding specificity and biological activities. These are the FGF1 subfamily, including FGF1 and FGF2; FGF4 subfamily, which includes FGF4, FGF5, and FGF6, the FGF7 subfamily made of FGF3, FGF7, FGF10, and FGF22, the FGF8 subfamily, which consists of FGF8, FGF17, and FGF18, as well as the FGF9 subfamily, which is made of FGF9, FGF16, and FGF20 (Ornitz and Itoh, 2015, **Table 2**). Finally, the FGF19 subfamily includes FGF19, FGF21, and FGF23 (Li, 2019). Heparin sulphate proteoglycan (HSPG) binding domains and N-terminal signalling peptides for secretion are common features shared by FGFs.

**Table 2:** Overview of Mammalian FGF subfamilies, receptor specificity and physiological functions.

The FGF Subfamilies	Ligands Human/mouse	Cofactor	Receptor Specificity	Major physiological function
**FGF1 subfamily**	FGF1/Fgf1	Heparin or heparin sulfate	All FGFRs	Adipose tissue homeostasis
	FGF2/Fgf2		FGFR1b,1c,2b,2c,3c,4	Wound healing and angiogenesis
**FGF4 subfamily**	FGF4/Fgf4		FGFR1c,2c,3c,4	Limb bud and heart development
	FGF5/Fgf5		FGFR1c,2c,3c	Hair follicle growth and development
	FGF6/Fgf6		FGFR1c,2c,3c,4	Muscle development and regeneration
**FGF7 subfamily**	FGF3/Fgf3		FGFR1b,2b	Inner ear and skeleton development
	FGF7/Fgf7		FGFR2b	Branching morphogenesis
	FGF10/Fgf10		FGFR1b,2b	Lung branching morphogenesis; inner ear, hair follicle, and limb development
	FGF22/Fgf22		FGFR1b,2b	Synaptogenesis
**FGF8 subfamily**	FGF8/Fgf8		FGFR1c,2c,3c,4	Brain, eye, ear, limb bud, kidney, and heart development
	FGF17/Fgf17		FGFR1c,2c,3c,4	Cerebellum and frontal cortex development
	FGF18/Fgf18		FGFR3c,4	Lung alveolar, bone, CNS, skeletal, and palate
				development
**FGF9 subfamily**	FGF9/Fgf9		FGFR1c,2c,3b,3c,4	Inner ear, gonad, and kidney development
	FGF16/Fgf16		FGFR1c,2c,3b,3c,4	Heart development
	FGF20/Fgf20		FGFR1c,2b,2c,3b,3c,4	Kidney, hair, teeth, cochlea, and central nervous
				development
**FGF15/19 subfamily**	FGF19/Fgf15		FGFR1c,2c,3c,4	Bile acid metabolism, gall bladder filling, lipid, and energy metabolism
	FGF21/Fgf21	β-Klotho	FGFR1c,3c,	Lipid, glucose, and energy metabolism, macronutrient preference, starvation response, insulin sensitivity
	FGF23/Fgf23	α-Klotho	FGFR1c,3c,4	Phosphate, calcium, sodium, and vitamin D homeostasis

In most cases, FGFs are secreted into the extracellular space *via* the classical secretory pathway. FGFs have been described to act both in an autocrine and paracrine fashion. They signal through their specific trans-membrane receptors consisting of five members, FGFR1-5. FGFRs bind their ligands with high affinity and various degrees of specificity (**Figure 2A**). All FGFRs has a single pass trans-membrane domain (TM) and two intracellular kinase domains (ICKs) except for the atypical FGFR5, which has no enzymatic activity (Trueb, 2011). The extracellular segments of FGFRs are composed of three Ig-like domains (IgGI-IgGIII), and between the first and second Ig-like domains is the acid-box domain that determines ligand-specificity. The ICKs harbor the catalytic activity of receptors as well as autophosphorylation sites, which interact with intracellular substrates (**Table 2**).

## The Role of FGFs in AKI

Many FGF ligands and their associated receptors are found to be highly expressed during kidney development. Genetic ablation and transgenic overexpression in mouse models, as well as exposure to exogenous FGF ligands, have established the critical regulatory roles of multiple FGF ligands in kidney development, particularly those that signal through FGFR1 and FGFR2 (Walker et al., 2016; **Figure 1B**). Some of the developmentally important FGFs/FGFRs expressed early during kidney development get re-expressed or activated in the regeneration phase after IRI (Villanueva et al., 2006; Tan et al., 2017). Currently, most of the FGFs reported to participate in AKI pathogenesis or reveal protective/therapeutic potentials are derived from FGF1, FGF7, and FGF19 subfamilies (**Figures 1A, B**), which we will be discussing in more detail.

## Multifaceted Roles of FGF1/2 in AKI

FGF1 (or Acidic FGF) and FGF2 (or basic FGF) are the prototypic members of the FGF family that have a similar broad range of biological activities. Both are *in vitro* mitogens for most of the ectodermal- and mesodermal-derived cell lines. Numerous studies have shown that intravenous administration of exogenous FGF1 or FGF2 can improve the physiological functions of different organs after IRI.

FGF1 is an autocrine/paracrine regulator known to act on cells from various organs and tissues, including liver, vasculature, and skin. FGF1 exerts significant beneficial effects in different organs after IRI (Fu et al., 1995; Cuevas et al., 2000; Chen et al., 2005; Wang et al., 2010). The skeletal muscle damage protection provided by FGF1 may arise from its ability to regulate extra-cellular and intra-cellular calcium ions concentrations (Fu et al., 1995). FGF1 can promote small intestine epithelial cell proliferation in IRI in rats and its activity is associated with the activation of ERK1/2. Intravenously administered FGF1 could also alleviate IRI-induced apoptosis in rat intestinal tissues (Weng et al., 2004). In other studies, FGF1 was reported to activate PI3K/AKT-mediated suppression of oxidative stress and inflammation, especially for diabetic nephropathy (Pena et al., 2017). FGF1 can protect hepatic and renal functions after intestinal IRI (Weng et al., 2004). Fu et al. showed that the protective effects of FGFs might originate from the non-mitogenic effects of FGFs at the early stage and the mitogenic effects at the late stage of tissue repair (Fu et al., 2004). Cuevas et al. demonstrated a role for FGF1 after acute kidney damage following IRI by inhibiting neutrophil infiltration (Cuevas et al., 1999).

FGF2 is another vital protein for ureteric bud formation, and also necessary for the induction of mesenchymal cells aggregation. The mesenchymal aggregates cannot give rise to epithelial cells in the absence of FGF2. In the adult mice, FGF2 exerted a protective function against IRI in several organs such as the retina, brain, spinal cord, heart, and intestine. FGF2 also has a beneficial role in renal IRI. The primary mechanism is *via* the activation of the PI3K/AKT and ERK1/2 pathways. The expression of FGF2 is not observed in the adult kidney during homeostasis, but a strong induction is observed in the regeneration phase of AKI. FGF2 reduces the expression of renal damage markers such as ED-1 and α-smooth muscle actin and participates in the regeneration process after ischemic acute renal failure (Villanueva et al., 2006). Xu et al. revealed the same results upon the ablation of the receptor FGFR2 in fibroblasts (Xu and Dai, 2017). Such genetic manipulation ameliorates kidney fibrosis after IRI in mice. On the other hand, inhibition of FGFR2 sensitize kidney damage and suppresses nephrogenic protein expression (Villanueva et al., 2008). Another study demonstrated that FGF2 protects against renal IRI by attenuating mitochondrial damage and proinflammatory signals (Tan et al., 2017). Functional studies have shown that FGF2 promotes proliferation on a variety of renal cell types, especially interstitial fibroblasts. FGF2 facilitates the epithelial to mesenchymal transition of tubular epithelial cells and contributes in the initial stage to an increase in the stroma population. The expression of FGF2 is increased in the process of stem cell treatment of AKI (Patel et al., 2012). We propose that both endogenous and exogenous FGF2 react to the transplanted stem cells after injury. The mechanism of stem cell therapy is not only the replacement of dead cells but more importantly, the role it plays through the secretion of growth factor as well as antiinflammatory molecules.

## Exogenous FGF10 Protects Against IRI

FGF7 and FGF10 were initially isolated as a keratinocyte growth factor 1 and 2, respectively. Both are expressed in the kidney, but unlike other FGFs, which are paracrine factors that are expressed exclusively in the mesenchyme. They interact primarily with the “b” isoforms of FGFR2 (FGFR2b), which is an alternatively spliced RNA isoform containing a unique domain (called IIIb) in the third IgG-like loop (Zhang et al., 2006). FGF7 and FGF10 have been documented to play an essential role in the development, growth, differentiation, and homeostasis of the muscosal lining of the urinary tract (Qiao et al., 1999; Walker et al., 2016).

Both FGF7 and FGF10 bind FGFR2b, but exert largely distinct physiological functions. FGF7 is implicated in both the induction of basal urothelial cell proliferation and the expansion of transitional epithelium. It is an efficient growth and differentiation factor during development and wound healing. FGF7 also has a significant effect on the kidney. Compared to wild type littermates, Fgf7 knockout mice display smaller kidneys with fewer ureteric buds and nephrons. Earlier reports showed that the expression of both FGF-7 and FGFR2b is induced and segregated between interstitial and epithelial cells in response to chemically-induced proximal tubular damage. The activation of this mesenchymal to epithelial paracrine signaling is implicated in the regulation of tubular repair process (Ichimura et al., 1996). Additionally, renal IRI is found to promote FGFR2 phosphorylation together with the selective upregulation of FGF7 and FGF2. Recombinant FGF7 administration can induce FGFR2 expression and promote bladder progenitor proliferation (Vinsonneau et al., 2010; **Figure 1B**), but no study on FGF7 and renal IRI has been published.

FGF10 is a multifunctional growth factor playing crucial roles in the development of multiple organs and tissues, including the kidney (Itoh, 2016). In contrast to the Fgf7-null mice, which do not display significant developmental abnormalities, the inactivation of Fgf10 in mice causes broad developmental defects including limb bud induction, lung as well as kidney agenesis (Ohuchi et al., 2000). Similar to FGF7, FGF10 also signals *via* interaction with its high-affinity receptor FGFR2b. Deletion of Fgf10 in mice led to kidney dysgenesis characterized by fewer collecting ducts and nephrons. Overexpression of a soluble dominant-negative FGFR2b isoform in transgenic mice revealed more striking defects, including renal aplasia or severe dysplasia (Celli et al., 1998). Intra-tracheal administration of FGF10 in rats with IR (Ischemia-Reperfusion) induced lung injury significantly diminished lung edema, the release of inflammatory cytokines, immune infiltration, and protein exudation. Activation of PI3K pathway has been reported to underlie FGF10-mediated protection against I/R-induced endothelial cell apoptosis and barrier dysfunction (Fang et al., 2014). More recent studies provided further evidence that exogenous recombinant FGF10 mediates protection against renal IRI through suppression of excessive autophagy and ER stress (Tan et al., 2018; Tan et al., 2020), which will be further discussed later.

## FGF23 as Biomarkers of AKI and CKD

FGF23 belongs to the FGF19 subfamily of endocrine FGFs which play important roles in interorgan crosstalk that governs a broad spectrum of metabolic homeostasis (Degirolamo et al., 2016; Li, 2019). Although there are a number of reports on the protective effect of FGF21 on the myocardial IRI, there has yet to be any published research directly addressing the role of either FGF19 or FGF21 in renal IRI. FGF23 is mainly produced by osteocytes and possibly osteoblasts. Significantly, it is dramatically increased in CKD and ESRD, and has been proposed as a biomarker for adverse outcomes in patients with CKD and ESRD (Isakova, 2012; Christov et al., 2019 **Figure 1B**). Similar to FGF19 and FGF21, FGF23 binding to FGFRs and the subsequent activation of FGF signal transduction requires a co-receptor, Klotho (Kurosu et al., 2006). The structure of FGF23 ternary complex together with α-klotho extracellular domain and the FGFR1c ligand-binding domain has recently been solved (Smith et al., 2014; Chen et al., 2018). FGF23 controls renal phosphate reabsorption, modulates the production of parathyroid hormone (PTH) and 1,25-(OH)2-vitamin D. It also participates in mineral homeostasis. FGF23 acts on the kidney to increase renal phosphate excretion and to decrease 1,25-dihydroxy vitamin D (1,25D) production (Li et al., 2019; Musgrove and Wolf, 2020).

FGF23 levels rose acutely in patients who underwent cardiac surgery and developed AKI, even before a significant increase in serum creatinine (Christov et al., 2013). In the current era of heightened awareness of the dire need for early diagnosis of AKI, FGF23 has been touted as a potential marker of the complex AKI syndrome (Leaf et al., 2017; Leaf et al., 2018). Shaker et al. reported that the change of plasma FGF23 concentrations at 24 hours after cardiac bypass surgery was associated with the severity of renal injury, whose sensitivity was 100%, and specificity was 97.1% suggesting that FGF23 could have a role as an early biomarker of AKI and predicts adverse outcomes among patients with established AKI (Shaker et al., 2018; Volovelsky et al., 2018). A recent prospective investigation in a large cohort of patients with CKD stages have identified elevated serum levels of interleukin-6, C-reactive protein, and FGF23 as independent risk factors for mortality in CKD (Munoz Mendoza et al., 2017). Circulating levels of FGF23 are increased in human AKI and CKD of various settings, and correlate to poor survival in patients across infants, children, and adults (David et al., 2017; Czaya and Faul, 2019). Therefore, serum FGF23 level is proposed to be an even more significant parameter than creatinine to assess the severity of the AKI (Christov et al., 2019).

Other studies demonstrate that tubule-derived FGF23 might amplify myofibroblast activation in AKI (Smith et al., 2017b). FGF23 is found to augment profibrotic signalling cascades in injury-primed renal fibroblasts *via* activation of FGFR4 and upregulation of the calcium transporter, a transient receptor potential cation channel. This function was independent of α-Klotho. Restoration of α-Klotho, as upstream regulators, can regulate the off-target effects of FGF23 (Smith et al., 2017a). Both FGF23 and α-Klotho have been proposed as prognostic biomarkers of AKI and also targets of therapeutic intervention for CKD or CVD after AKI. The effects of different FGF family members in the context of AKI is summarized (**Table 1**).

## FGFRs Involved in AKI

The FGF ligands signal through four receptors (FGFR1-4) and an atypical, kinase inactive FGFR5 or FGFRlL (Ornitz and Itoh, 2015). Some of FGFs/FGFRs are important during normal kidney development and also during postnatal repair. FGFs stimulate kidney cell fate determination, migration, and differentiation during organogenesis (Bates, 2011; Trueb et al., 2013) and regulate the proliferation, mobilization, and regeneration during repair after injury (Strutz, 2009; Gallegos et al., 2019).

FGFRs elicit different functions in mammalian development and diseases. Conditional knockout of Fgfr1 in ureteric bud and metanephric mesenchyme did not lead to any kidney development defects (Revest et al., 2001; Poladia et al., 2006). Ablation of Fgfr2 led to the formation of a smaller kidney with fewer nephrons (Sims-Lucas et al., 2011). The combined inactivation of Fgfr2 and Fgfr1 knockout in mice led to severe kidney aplasia (Sims-Lucas et al., 2011). General knockout of Fgfr3 and Fgfr4 did not affect early kidney development (Colvin et al., 1996; Weinstein et al., 1998). It has been previously reported that a given FGF ligand can bind multiple receptors and that conversely, the same receptor can bind different ligands, therefore allowing a very complex set of biological activities downstream of FGF/FGFR activation (Li, 2019). FGFR1, for example, can bind to most of FGFs except FGF7 and FGF18. FGFR2b, on the other hand, binds only FGF1 and the members of the FGF7 subfamily. Due to potential redundancy in FGFRs, their role in acute kidney injury is still unclear. However, exogenous FGF ligands, through the activation of the FGFR signalling pathway, are protective against renal damage. During IR-induced acute renal injury, increased expression of FGF activates FGFR phosphorylation and recruitment of FRS-2 and PLC-γ. The classical downstream PI3K/AKT and MAPK signalling pathways are subsequently activated to control cell proliferation, differentiation and apoptosis as well as cell migration and other processes. This leads to decreased renal injury caused by IRI (**Figure 2A**).

Although IRI mainly affects renal tubular cells, IR-induced pathogenic process and its repair involve interactions between interstitial cells, infiltrated inflammatory cells and epithelial cells. Interestingly, a recent study using a mouse model with fibroblast-specific ablation of Fgfr2 gene indicated that FGFR2 expression in fibroblast may contribute to kidney fibrosis after IRI through promoting renal fibroblast activation and proliferation (Xu and Dai, 2017).

## Impact of FGFs on Pathophysiology of IRI

IRI represents a frequent underlying cause for both ischemic heart disease and AKI. Compared to the numerous publications on a broad spectrum and relatively extensive research on FGFs in ischemic heart injury, only a limited number of reports are available on FGFs (mainly FGF2, FGF10, and FGF23) in renal IRI and AKI. Additionally, most of FGF23-related studies focus on its role as a key metabolic regulator and an injury or prognosis biomarker. Consequently, FGF23 is a proinjury factor rather than a protective or therapeutic agent for AKI. Therefore, our following discussions will only focus on FGF2 and FGF10, both of which exhibit potent protection against IRI and therapeutic potential *via* impacting several key pathophysiological mechanisms.

## FGF2 Protects Against IR-Induced Tubular Cells Death

Acute cell death of proximal renal tubules and inflammatory response of both innate and adoptive nature are hallmark features of IRI. Apoptosis plays an important role in the IR-induced pathogenesis and is a validated parameter to evaluate the cellular damage induced by ischemia. The protective role of FGF2 against IRI is well documented for ischemic myocardial infarction. Studies in genetically engineered mouse models established the antiapoptotic effect of endogenous FGF2 toward ischemic cardiac injury. FGF2 also exerts a positive impact on the repair process that may involve its activation of both MAPK and PKC pathways (House et al., 2003; House et al., 2005; House et al., 2007). Furthermore, under cardiac IRI conditions mimicking clinical acute myocardial infarction, endogenous FGF2 is considered an essential acute cardioprotective factor and a longer term proangiogenic factor (House et al., 2015). The protective effect of FGF2 against IRI and in promoting repair was also appreciated in renal IRI (Villanueva et al., 2008; Tan et al., 2017). Interestingly, FGF2 is found to be expressed early during kidney development, and gets re-expressed upon IRI and participates in the recovery process by promoting the expression of morphogen proteins to accelerate the repair process in the kidneys (Villanueva et al., 2006). Additionally, treatment with antisense oligoes targeting FGFR2 led to a significant increase in tubular TUNEL positive cells and expression of damage markers in an AKI model, whereas the expression of morphogenic proteins and cellular mitosis was inhibited (Villanueva et al., 2008). Our study demonstrated that exogenous recombinant FGF2 also exhibited robust protection against IRI and significantly improved animal survival in a rat IRI model (Tan et al., 2017).

Under hypoxic conditions, FGF2 may alleviate oxidative stress and IR-induced mitochondrial DNA damage and proapoptotic alteration of Bcl2/Bax expression and caspase-3 activation. The remarkable protective effect of FGF2 owns, at least in part, to its ability to preserve the integrity of the mitochondrial ATP-sensitive potassium channel (Tan et al., 2017). It is worth noting that, besides the major FGF2 protein isoform (18 kD, low molecular weight), there exist at least 4 other isoforms of higher molecular weight, which are reported to exert different or even opposite effects on apoptosis (Kardami et al., 2007; Liao et al., 2010; Manning et al., 2013) *via* different mechanisms. Besides apoptosis, other types of cell death such as necrosis, necroptosis, pyroptosis, ferroptosis, have been also been implicated to underlie the tubular cell damage, the potential role of FGFs on these additional pathways and the their interplay remain to be characterized (Xu and Han, 2016; Han and Lee, 2019; Hu et al., 2019). On the other hand, renal recovery from AKI requires the replacement of injured cells by new ones that can restore tubular epithelial integrity. In this regard, FGF2 may also facilitate the repair process of IRI, as post-IRI administration of FGF2 also exhibited effective protection of IRI and improved animal survival (Tan et al., 2017). The results collectively indicate that FGF2 has promising clinical potential for the prevention and treatment of IR-related AKI.

## FGF10 Inhibits Excessive Autophagy and ER Stress

Autophagy is an evolutionarily conserved pathway that leads to lysosomal degradation of cytoplasmic substrates, such as damaged organelles and cytoplasmic proteins. The autophagic response is triggered under various stress conditions including nutrient starvation, hypoxia, and growth-factor deprivation, as well as ER stress and oxidant injury, most of which are involved in the pathogenesis of AKI. Knockout of Atg5 or Atg7 in proximal tubule led to accumulation of deformed mitochondria, ubiquitin-positive inclusion bodies increased apoptosis and worsened renal dysfunction upon IRI suggesting a role of autophagy to the normal homeostasis of the kidney and renoprotective effect in IR injury (Jiang et al., 2010). However, some studies also report that autophagic response exacerbates renal IRI (Kaushal and Shah 2016; Tan et al., 2018). Therefore, it is likely that autophagy may exhibit both protective and detrimental properties in renal IRI, depending on the duration, the phase, and even the extent of the IRI.

FGF can inhibit autophagy through the mTOR pathway and block differentiation during organogenesis (Zhang et al., 2012; Cinque et al., 2015). Different FGFs may have distinct activities to regulate autophagy. FGF21 protects cardiomyocytes by promoting autophagic ﬂux with hypoxia/reoxygenation injury (Ren et al., 2019). On the other hand, FGF2 and FGF10 can alleviate IRI by suppressing excessive autophagy *via* PI3K/AKT and MAPK signaling in the kidney and other organs (Wang et al., 2015; Sun et al., 2018; Tan et al., 2018, **Figure 2B**). Based on the analysis of renal tissues for their LC3, Beclin-1 and SQSTM1 expression and localization, FGF10 treatment was found to significantly suppress autophagic phenotype, which was highly activated during IRI, whereas co-treatment of FGF10 with Rapamycin partially reversed such renoprotective effect, suggesting the involvement of mTOR pathway in the process. This study established that exogenously administered recombinant FGF10 is protective against IR-induced functional and tissue damage to the kidney, at least partially through mitigating excessive autophagy.

Recent studies further showed that IRI is accompanied with excessive activation of ER stress, which is involved in hypoxia injury-induced apoptosis of renal tubular epithelial cells. ER, a specialized organelle for protein synthesis, folding and trafficking, is highly sensitive to the intracellular microenvironment changes. Hypoxia and oxidative stress are intrinsic to IRI, which disturb ER functions and lead to impaired protein folding (Walter and Ron, 2011; Cao and Kaufman, 2014). Excessive accumulation of unfolded proteins activates Unfolded Protein Response (UPR), a cellular stress response mechanism to improve the protein folding efficiency while reducing mRNA translation along with protein expression (Schuck et al., 2009). Although ER stress plays an important role in cell growth and differentiation, excessive activation of ER stress and UPR can activate apoptotic signaling (Tabas and Ron, 2011; Hetz, 2012), which is mainly mediated by C/EBP homologous proteins (CHOP), a master regulator of maladaptive ER stress-induced apoptosis. FGF10 effectively alleviated IRI evoked expression of ER stress-related proteins in the kidney including CHOP, GRP78, XBP-1, and ATF-4 and ATF-6, which may contribute to inhibit IR-induced activation of proapoptotic caspase-3 and Bax expression. Results from IRI model *in vivo* and *in vitro* cell culture experiments together indicate that FGF10 attenuates renal tubular epithelial cell death *via* inhibiting excessive ER stress, which is, at least in part, mediated by MEK-ERK1/2 signaling pathway (Tan et al., 2020). Therefore, FGF10 contributes to restore the balance between the adaptive pathway and the apoptotic pathway of UPR by inhibiting excessive ER stress.

## Role of FGFs on Inflammation

The inflammatory response is an integral component in the initiation and exacerbation of AKI. Although a crucial element of the repair process, excessive activation of inflammatory signals and cytokine secretion may impose further damage to renal parenchyma cells. FGFs may exhibit different impacts on the inflammatory process of IRI. Contrary to the protective effects of FGF2 and FGF10 against IRI/AKI, FGF23 appears to be a deleterious factor.

Under normal physiology, FGF23 is mainly produced in bone by osteoblasts/osteocytes in response to local and systemic factors and targets the kidney to create multiple endocrine networks (Bergwitz and Juppner, 2010). Although some studies suggest that FGF23 interacts with the immune system, it is not clear whether FGF23 directly regulates immune cell functions or indirectly impacts immune responses through FGF23 regulation of 1,25-dihydroxyvitamin-D. FGF23 is increased in CKD. Macrophages do not regularly express FGF23 or α-Klotho, but in acute inflammation, FGF23 stimulates proinﬂammatory responses in M1 macrophages and blocks the transition to M2 macrophages (**Figure 1B**). In addition, FGF23 is proposed to activate FGFR2 in polymorphonuclear leukocyte to directly decrease their recruitment. Given the link of increased serum FGF23 to various tissue injuries, as well as evidence that the sources of FGF23 and control of its production in AKI and CKD differ from those in the physiologic conditions, mediators of inflammation contributing to elevated FGF23 have recently been proposed as potential drug targets, in addition to repurposing existing strategies to target FGF23 (Musgrove and Wolf, 2020).

High mobility group box 1 protein (HMGB1) is a highly conserved nuclear protein that functions as an architectural chromatin-binding factor and regulator of gene transcription. HMGB1 assumes diverse roles as an immuno-modulator in the form of a cytokine molecule or as nuclear chromatin and transcription regulator. HMGB1 can be activated and gets released from damaged parenchymal cells as a sterile inflammatory molecule and a major damage-associated molecular pattern (DAMP). Translocation of HMGB1 from the nucleus to the cytoplasm and subsequent release to the extracellular milieu is reported to promote inflammatory response *via* the activation of TLRs (Yang et al., 2010). HMGB1-TLRs pathway has long been recognized as an essential and early mediator in renal IRI and an attractive target of AKI and other disease therapies (Li et al., 2011; Zhang et al., 2016; Vijayakumar et al., 2019).

Recent efforts to explore the potential protective effect of exogenous FGFs on renal IRI illustrated that, besides promoting proliferation and inhibiting apoptosis, both FGF2 and FGF10 effectively inhibited IRI-induced release of HMGB1 from the nucleus to the extracellular domain, which is associated with a marked decrease in the expression of inflammatory cytokines such as TNF-α, IL-1β, and IL-6 following IRI (Tan et al., 2017; Tan et al., 2018, **Figure 2B**). This novel function of FGFs on the inflammatory cascade may be related to the inhibition of HMGB1-mediated TLR2 and/or TLR4 signaling (Leemans et al., 2009; Wu et al., 2010; Chen et al., 2017). It has been reported that FGF10 ameliorates cerebral ischemia injury *via* inhibiting NF-κB-dependent neuroinﬂammation and activating PI3K/AKT survival signalling pathway (Li et al., 2016). Whether the inhibition of HMGB1 and associated inflammatory cytokine release by FGFs is due to their protective effect against renal damage and therefore less DAMP release, or through some other mechanism(s) remains to be further elucidated.

## Conclusion and Prospect

Extensive research in the past years have established FGFs as vital regulators in tissue repair and regeneration, as well as in metabolic homeostasis. Along with advances in our understanding of FGF biology and their regulation of various pathophysiologic processes comes the inspiration of harnessing their power for potential disease therapies.

In this review, we summarized the available data on the FGFs that have shown promising features related to IRI either as a preventative/therapeutic agent or a biomarker. We highlighted some functional and biological aspects that constitute the promising features of FGFs, particularly FGF2 and FGF10, in averting IR-induced tubular cell death and inhibition of overt inflammatory response, which may all contribute to their net beneficial effect on reducing the IRI and promoting recovery.

Admittedly, the exploration of FGFs as preventative or therapeutic agents in the management of clinical IRI is still in its rudimentary stages. Most of the published work has been conducted in pre-clinical settings. These widely used animal models, including the bilateral IR that is considered to best resemble clinical AKI, still have certain limitations in fully mimicking human AKI, which can be caused by numerous and complicated clinical conditions. Additionally, the underlying pathophysiology of IRI and how FGFs impact key biological and pathophysiological processes, such as apoptosis, autophagy, ERS, and oxidative stress, remains poorly understood. Future research should encourage more clinical-based and patient-oriented studies. Moreover, the utilization of advanced genetic animal models will also be instrumental in elucidating the disease mechanisms. This will allow for the labeling and tracing of specific cell populations to gain deeper insights about the effects of FGF. Collectively, these approaches, combined with proteomic and genomic technologies, will better delineate FGF signalling targets and support the therapeutic potential of FGFs toward AKI.

## Author Contributions

J-SZ and L-CD conceived the study. L-CD, TA, SB, and J-SZ drafted and revised the manuscript.

## Conflict of Interest

The authors declare that the research was conducted in the absence of any commercial or financial relationships that could be construed as a potential conflict of interest.

The handling editor is currently organizing a Research Topic with several of the authors SB, J-SZ, and confirms the absence of any other collaboration.

## Abbreviations

AKI, acute kidney injury; AKT, protein kinase B; CKD, chronic kidney disease; CSA-AKI, cardiac surgery-associated AKI; DAMP, damage-associated molecular pattern; ERK, extracellular signal-regulated kinase; ER, endoplasmic reticulum; ESRD, end-stage renal disease; FGF, fibroblast growth factor; FGFR, fibroblast growth factor receptor; FRS-2, fibroblast growth factor receptor substrate 2; HMGB1, high mobility group protein 1; HSPG, heparin sulphate proteoglycan: ICK, intracellular kinase domain; IL-1β, Interleukin-1 beta; IL-6, Interleukin-6; IR, ischemia reperfusion; IRI, ischemia reperfusion injury; MAPK, mitogen-activated protein kinase; NF-κB, nuclear factor kappa-B; PI3K, phosphatidylinositol-4,5-bisphosphate 3-kinase; PLCγ, phospholipase C gamma; ROS, reactive oxygen species; TGF-β, transforming growth factor beta; TLR2; toll-like receptor 2; TLR4, toll-like receptor 4; TM, trans-membrane domain; TNF-α, tumor necrosis factor alpha; UPR, unfolded protein response.
